# Caregivers’ experiences and practices for malnourished children undergoing tuberculosis treatment in Eswatini

**DOI:** 10.4102/hsag.v29i0.2349

**Published:** 2024-04-05

**Authors:** Bhekisisa S. Tsabedze, Debbie S.K. Habedi

**Affiliations:** 1 Department of Health Studies, Faculty of Public Health, University of South Africa, Pretoria, South Africa; 2 Eswatini Ministry of Health-National AIDS Program, Mbabane, Eswatini

**Keywords:** caregivers, experiences, malnourished children, practices, tuberculosis treatment

## Abstract

**Background:**

Eswatini is one of the countries affected by malnutrition and tuberculosis (TB) and some cases remained untreated. These two conditions are major public health problems.

**Aim:**

This study aimed to explore and describe caregivers’ experiences and practices of children’s nutrition during treatment.

**Setting:**

Baylor College of Nursing Children’s Foundation – Eswatini (BCMCF-SD).

**Methods:**

A qualitative study following a narrative design used purposive sampling to identify 12 caregivers of malnourished children and informed consent obtained. In-depth interview used semi-structured interview guide and digital voice recorder. Field notes were taken, transcribed, translated and analysed using NVivo version 11.

**Results:**

Two themes emerged as home’s nutritional situation and health facility’s nutritional support. The study found that most of the caregivers gave children unbalanced diet, while those less than a year were mixed-fed. Some caregivers reported experience of lost breadwinners, unemployment and high number of children than what the family could afford. The caregivers’ practices around food by prescription included inadequate supply of the ready-to-use therapeutic food and sharing of prescribed food supplies with other healthy children.

**Conclusion:**

During treatment, children’s caregivers need short health education and support. The Ministry of Health in Eswatini should consider using some comic books to guide that. Moreover, upscale vocational training promotes entrepreneurship and agricultural activities.

**Contribution:**

Association of malnutrition and TB outcomes has provided evidence-based information for more comprehensive integration between nutrition programmes and tuberculosis programmes. The study’s findings contributed to the growing body of knowledge about the association between malnutrition and diagnosed drug-susceptible TB among children aged from 0 – 15 years.

## Introduction

Globally, the combination of malnutrition and tuberculosis (TB) poses a major challenge in the treatment and care of children diagnosed with TB (Munthali et al. 2017). In 2018, the World Health Organization (WHO 2018) estimated that 1.1 million children were diagnosed with TB globally, which leads to about 205 000 deaths. Globally, children under 15 years represent approximately 12% of new TB cases, but 16% of the estimated 1.4 million deaths. This higher share of mortality highlights the urgent need to develop strategies to improve case detection in this age group and identify children without TB disease who should be considered for TB preventive treatment. One such strategy is systematic screening for TB in high-risk groups (Vonasek et al. 2020).

Eswatini is a small landlocked country between South Africa and Mozambique and usually referred to Swaziland. This country has an employment rate of 41% and a poverty rate estimated at 63% (Dhemba & Nhapi 2020). The country also has low TB case notifications estimated at 6% among children less than 15 years. Thus, the unnotified 94% of children with TB disease are predisposed to malnutrition because of delayed or poor TB case notification (WHO 2018). However, if the children with TB disease are notified early, they would be enrolled in nutrition therapy, while getting the TB treatment.

Dlamini and Tlou (2022) conducted a study on prevalence and associated risk factors of chronic malnutrition among children under 5 years in Eswatini. The findings revealed that a large number of children under the age of 5 years still fail to achieve their growth potential, as reflected by the prevalence of malnutrition. The findings also indicated that a low birth weight, mothers’ education and the child’s age were significant risk factors associated with chronic malnutrition. Despite the high TB-related mortality, childhood TB remains neglected among healthcare workers because of its difficulty to be diagnosed and treated, successfully (Amanullah et al. 2019). The integrated childhood TB into guidelines for the management of acute malnutrition in high-burden countries further reported that even though the underlying mechanism of the association between malnutrition and TB is unknown, it is reported that about 45% of TB deaths in children are aggravated by malnutrition (Patel & Detjen 2017). However, challenges in Eswatini’s TB response remain. According to WHO (2023), the kingdom continues to have a high burden of TB, with 319 new cases of TB reported per 100 000 people in 2020 (Centers for Disease Control and Prevention 2022). United Nations International Children’s Emergency Fund (UNICEF 2018) states that for every child in Eswatini it is reported that stunting, which is a sign of chronic malnutrition, is a serious problem, with the under-five population estimated at 150 643 (13.3%) of the total population, 25% of children under the age of five being short for their age, estimated 60, 257 children are stunted (https://www.unicef.org/eswatini/nutrition).

Malnutrition is estimated to contribute to more than half of deaths in children worldwide (Vonasek et al. 2020); child malnutrition was associated with 54% of deaths in children in developing countries. Poverty, disease and illness remain the major contributors to this situation. Bain et al. (2013) cited in Djoumessi (2022) and Muller and Krawinkel (2005) argued that malnutrition is globally the most important risk factor for illness and death, with hundreds of millions of pregnant women and young children particularly affected. They defined malnutrition as deficiencies in iron, iodine, vitamin A and zinc in developing countries. Although treatment protocols for severe malnutrition have in recent years become more efficient, most patients (especially in rural areas) have little or no access to formal health services and are never seen in such settings. Interventions to prevent protein-energy malnutrition range from promoting breastfeeding to food supplementation schemes, whereas micronutrient deficiencies would best be addressed through food-based strategies such as dietary diversification through home gardens and small livestock (Muller & Krawinkel 2005).

Literature reported that caregivers have an essential responsibility that they play, which results in the successful treatment of children diagnosed with TB, especially when the child and the caregiver reside together (Galli et al. 2016). The place of residence for the children and caregiver is a significant determining factor for the child’s TB outcome (Kebede, Taye & Matebe 2017). Care for children with severe acute malnutrition (SAM) at the facility and community level is provided by healthcare staff and by mothers and other carers. These caregivers have a unique insight into factors affecting survival of children with SAM. Little research has been carried out to understand the perspectives and recommendations of these different caregivers on providing quality care (Fergusson et al. 2010). Tornu et al. (2022) attest that ill children living with TB require considerable parental or guardian care to survive. Mothers typically play the traditional role of primary caregivers for children in most settings, including Ghana. As such, it is important to learn if and how the mothers’ burden of care associated with their caregiver role changes when their children fall ill with TB and must be nursed back to health. Caregivers of children diagnosed with malnutrition and drug-susceptible TB are vital stakeholders when it comes to safeguarding the health, well-being and overall survival of the children whom they care for.

The healthcare system should be designed in a way that it can provide individualised caregiver support to make sure that the children get comprehensive care. According to WHO, there is a great need that healthcare workers (HCWs) should seek to offer individualised care for the various caregivers (WHO 2016). The interventions should have evidence of recognising the various individual social needs as organised by the specific health conditions of the caregiver (Schulz & Eden 2016). As much as there are caregiver support groups for human immunodeficiency virus and acquired immune deficiency syndrome (HIV and AIDS) at Baylor College of Medicine Children’s Foundation (BCMCF) in Eswatini, the psychosocial support (PSS) provided to caregivers of malnourished children diagnosed with TB is lacking. Lopez-Ejeda et al. (2019) conducted a study on review of operational experiences in delivering SAM treatment through community health platforms and identified the caregivers’ awareness of the condition and/or availability of services, distance and high opportunity costs as the most important barriers affecting coverage of outpatient, facility-based treatment for SAM.

Tuberculosis is the most popular respiratory infection ranking above HIV globally (WHO 2019). Furthermore, the WHO report published in 2020 reported that malnutrition was associated with more than half of all child deaths in developing countries in the 1990s (Djoumessi 2021). Infectious diseases are the immediate cause of death for most of the millions of children under the age of 5 years who die each year in the developing world including Eswatini. However, the risk of death from these diseases is significantly higher for children who are hungry or malnourished (WHO 2021).

Moreover, there is no known study conducted before that has explored and described the experiences and practices of caregivers regarding malnourished children on TB treatment in Eswatini. However, understanding the caregiver’s experiences and practices will enable appropriate, timely individualised caregivers’ support as they take care of the children diagnosed with TB. The researcher conducted data quality assurance (DQA) for BCMCF in 2016, which indicated that there was a steady increase in the death rate for children taking TB treatment (Baylor College of Medicine Children’s Foundation – Swaziland 2015–2016). Baylor College of Medicine Children’s Foundation-SD’s TB register data further indicated that 7 out of the 40 (17.5%) children died from drug-susceptible TB. In comparison, in 2014, there were 4 TB-related fatalities occurred out of 37 children diagnosed with drug-susceptible TB. Five children had poor TB outcomes ranging from unevaluated cases, failing TB treatment, missing to follow-up and TB relapse. It was also gathered from the TB register that ready-to-use therapeutic food and food by prescriptions were supplied to most of the children along with their TB treatment. However, the nutritional status of the children remained poor when calculated using a z-score, irrespective of the nutritional support they received from the health facility. Thus, it remained crucial that the authors conducted a study that explored the experiences and practices of the children’s caregivers. With growing attention globally to the childhood tuberculosis epidemic after decades of prevalence, and with burden of malnutrition in most of the world’s countries, the collision of these two diseases is an important focus for improving child health.

## Purpose of the study

The purpose of this study was to explore and provide a description of the experiences and practices of the caregivers regarding paediatric nutrition during TB treatment at BCMCF in Eswatini.

## Research question

What are the experiences and practices of the caregivers regarding paediatric nutrition during TB treatment at BCMCF in Eswatini?

## Research methods and design

The study was conducted using a qualitative research approach following a narrative design (Nigar 2020) among caregivers of malnourished children on TB treatment at BCMCF located in the Hhohho region of Eswatini. BCMCF is an extension of Baylor College of Medicine (BCM) in Texas in the United States. The participants were recruited through purposeful sampling from the period of 01 April 2018 to 30 June 2018 during their routine clinical visits at BCMCF, Eswatini.

The inclusion criteria consisted of willingness to participate in the study, being a caregiver of the child less than 15 years, diagnosed of malnutrition, while on TB treatment at the study site. Informed consent was obtained from all eligible selected caregivers. Post signing the informed consent, qualitative data were collected using semi-structured interviews in a room designated for the study interviews only, away from other destructions. A voice recorder, black pen (ink) and sheets of field notes were used to collect nonverbal clues, sociodemographic characteristics and the experiences of the caregivers about the nutrition of their children during TB treatment at BCMCF in Eswatini.

According to Sutton and Austin (2015), field notes are an essential part of the data. The interview guide was formulated in English and translated to SiSwati by the researcher. It consisted of the sociodemographic section with eight dichotomous questions and seven semi-structured questions inclusive of probes. Each interview took about 40–60 min.

After interviewing 12 eligible participants, data saturation occurred, as there was no additional information the researcher could obtain from further interviews. The themes and categories became repetitive and redundant (Sunders et al. 2018). The collected data were transcribed concurrently with the data collection. Transcription was transcribed using the participants’ language and later translated to English by the researcher. The qualitative data analysis involved rechecking the transcripts against the voice record and the development of themes and categories through data coding.

After rechecking the transcript against the audio-recorded data, the researcher translated transcripts written in SiSwati to English using Microsoft Word. The well-organised data were further analysed using NVivo version 11. The researcher identified similar statements and grouped them under one relevant theme in NVivo software, which helped in categorising emerging themes. The audio records were transferred to a laptop and an earpiece was used to listen to each interview while comparing it with the transcript. All necessary corrections were made along with the audio.

### Trustworthiness

The researcher ensured scientific rigour through trustworthiness. Trustworthiness ensures that the findings can be trusted through the application of quality criteria (Korstjens & Moser 2018). In this study, credibility was ensured through persistent observation to identify the characteristics that were relevant to the problem. The study team evaluated the data for each of the participants. The participant’s characteristics, behaviour and experience were described in detail to ensure transferability of the findings to another similar setting or population. In addition, existing literature was used to guide the findings of the study. All the research steps were described from the ignition stage to the end of the study. To ensure confirmability, the exact words of the participants were depicted, and the records of the research will be kept for up to 5 years post the study. Approved protocol was used to guide the study. The interview guide enabled the study participants to be able to examine their experiences and practices while the researcher observed their reflexivity (Korstjens & Moser 2018).

### Ethical considerations

Throughout the study, the researcher adhered to research ethics as guided by the Belmont Report of 1979 and the Declaration of Helsinki ethical principles. These included respect for study participants, fairness, beneficence, informed consent and no harm. Arifin (2018) continues to state that it is essential to apply research ethics to protect the research participants from harm. Thus, this study obtained ethical clearance (HSHDC/611/2017) for the University of South Africa (UNISA), Higher Degrees Committee and the Swaziland Scientific and Ethics Committee (SSEC), BCMCF Ethics Committee and the Basic Research Assurance International Network (BRAIN) based in the United States. The researcher respected the autonomous decision of the participant to be a participant through the provision of noncoercive informed consent. All the participants were treated with fairness as the research observes the principle of justice and their information was treated with the same standard of confidentiality. Moreover, other ethical principles such as anonymity, nonmaleficence and beneficence, informed consent and justice were also upheld throughout the study. Lastly, all risks and benefits were explained to the study participants.

## Results

The demographic characteristics of the study participants are included in the results and the two main themes emerged during the data analysis. The main themes were presented in categories and subcategories. The verbatims for participants are indented.

### Characteristics of the participants

Only females with an age range between 18 years and 65 years and having malnourished children on TB treatment were recruited for the semi-structured interviews. Most of the participants were aged between 40 and 49 years, while there was only one that was aged between 50 and 65 years. The participants comprised five widows, five married mothers and two single mothers. Most of the participants were unemployed, while only one was self-employed and three were employed. All participants reported that they had at least one employed family member and 11 participants reported that they had more than three children to feed. Table 1 summarises the themes, categories and subcategories that were identified.

**TABLE 1:** Children caregivers’ experiences and practices.

Themes	Category	Subcategories
1. Home’s nutritional situation	1.1. Nutrition for the child	1.1.1. Lack of balanced diet
		1.1.2. Mixed feeding during the breastfeeding period
	1.2. Social environment	1.2.1. Loss of loved ones
		1.2.2. Low socioeconomic status
		1.2.3. Having more children, which the family could not afford
2. Health facility’s nutritional support	2.1. Food by prescription	2.1.2. Ready-to-use therapeutic food supplies
		2.1.2. The food by prescription packages
		2.1.3. Sharing of the food supplies
	2.2. Health education	2.2.1. Health education too lengthy

### Theme 1: Home’s nutritional situation

The home’s nutritional situation emerged as the initial theme, with only two categories and five subcategories identified for this theme. The categories included the nutrition of the child and the social environment.

#### Category 1.1: Nutrition for the child

Two subcategories were identified, namely a lack of a balanced diet and mixed feeding during the breastfeeding period. The subcategories reflect the participants’ views and feelings regarding the nutrition provided to the children during the period of TB treatment.

**Subcategory 1.1.1: A lack of balanced diet:** Three of the participants reported that they lacked a balanced diet in their home, and they had the assumption that the children developed malnutrition because of the lack of a balanced diet. The exact words of the participants were quoted as follows:

‘The child could rarely get a balanced diet. The only food I was able to give to her was porridge. I was affording beans and sour milk maybe twice a week.’ (Participant 7)

‘I cannot lie, the nurses were telling me to give breast milk only to the baby. However, it was very difficult for me to adhere to their instructions.’ (Participant 1)

‘… when the child start crying, I was giving him some sour porridge, along with breast milk.’ (Participant 11)

**Subcategory 1.1.2: Mixed feeding during breastfeeding period:** Also, three of the study participants reported that they were taking care of breastfeeding children (Participants 4, 9 and 2). Two participants also mixed fed their children. They then had an assumption that the children were not well nourished, as some of the mothers used to work until late and arrive home very tired. Their exact words were as follows:

‘I raised this child by myself. Her mother was a drunkard. She used to knock off and go to drink alcohol and come home very drunk, at times unable to breastfeed the child.’ (Participant 4)

‘I do not think the child was getting enough food. As much as her mother was breastfeeding her and giving formula feeding, there was a time whereby the mother could lack money for formula feeding. Thus, I used to feed that child with family meal when she is at work. The situation became worse after the death of the mother.’ (Participant 9)

One of the caregivers was not a biological parent of the child, and she reported that the child’s biological parent was addicted to alcohol. Her exact words were as follows:

‘Raising the child alone while her mother was busy with alcohol was very hectic for me. The child could go for several days without being breastfed.’ (Participant 2)

#### Category 1.2: Social environment

Three subcategories were further identified from this category as shown in Table 1. The caregivers felt that the social environment was exposing the children to health hazards, such as TB reinfection. Other social events that were identified included the death of a parent, a lack of food because of unemployment, and a higher number of children than what the family could afford.

**Subcategory 1.2.1: Loss of loved ones:** One of the participants reported that the biological caregiver of the child died about a month or two before the date the child was diagnosed as TB positive. The participant continued to report that the child was deprived of nutrients from the breast milk after the death of the biological caregiver. The participant’s words were captured as follows:

‘As I have already said, most of the children in the village were diagnosed with TB post the death of either parent. Thus, we suspect that they get an imbalanced diet after the loss of their significant other, especially the breadwinners.’ (Participant 2)

**Subcategory 1.2.2: A low socioeconomic status:** All the participants mentioned unemployment as a contributing factor to the lack of adequate food for their families. They were sometimes running out of money to buy formula feeds for two children less than 12 months old, which resulted in families starting to feed the children with starch only. The views of the participants were documented as follows:

‘I can say the source of everything is unemployment. It is not easy to find a job in this country. So, so how can one survive?.’ (Participants 7)

‘Due to the high unemployment rates, people are squat in one room and the TB infection spreads, even more, faster.’ (Participant 4)

**Subcategory 1.2.3: Having more children, which the family could not afford:** Only one family reported having a single child (Participant 10) and the rest had three and more. The participants expressed that they could not afford to feed the children in their families. The participants reported that some additional children were members of the extended family, while some were from their neighbours (Participants 3, 4, 5, 7 and 11). Some of their words were quoted as follows:

‘Where I stay there are a lot of children from the neighborhood homestead. They come and play with my kids and then I have to feed all of them. Some of these children are very poor and vulnerable.’ (Participants 3, 4, 5 and 7)

One of the participants (Participant 11) who has five children reported that in her house, she used to feed seven more children from the extended family. She expressed herself as follows:

‘We are one family; so, the children cannot understand why we do not give them the food. We also had an assumption that these food packages would prevent the children from TB disease.’ (Participant 11)

### Theme 2: Health facility’s nutritional support

Health facility’s nutritional support emerged as the second theme of the study. The study participants shared their feelings regarding the support that they received from the health facility. Two categories were identified under this theme, which were food by prescription and health education.

#### Category 2.1: Food by prescription

The food by prescription can be defined as a nutritional therapy that was given to the children for nutritional support during the period of TB treatment. The category for food by prescription had three subcategories identified, which were ready-to-use therapeutic food supplies, food by prescription supplies and sharing of the food supplies.

**Subcategory 2.1.1: Ready-to-use therapeutic food supplies:** The food supplies were prescribed for most of the caregivers to give to their children. The caregivers reported that they used to share the ready-to-use therapeutic food with other children within their extended families, as they were also hungry, and this resulted in some children running out of ready-to-use therapeutic food stock before the expected period (Participants 2 and 4). The words of the participants were as follows:

‘As we used to paste bread with it in the morning. It was bound to be finished before the scheduled date.’ (Participants 2)

‘There was a very high demand for the food packages, such that it used to get finished before some of us receive it.’ (Participants 4)

**Subcategory 2.1.2: The food by prescription packages:** The food by prescription packages was given to the children who were aged 12–15 years, while those less than 12 years were provided with Plumpy’Nut. The packages for these children included 2 L of cooking oil, 5 kg of beans and one bag of maize. Almost all the caregivers of the children were eligible for food by prescription, but some participants reported that at times the packages were incomplete. They reported that one could find that it misses one of the packages like beans or cooking oil, if not both (Participant 11). The following statement depicts the words of one of the participants:

‘For me, there was no day whereby I went away without my prescribed food from the health facility. However, there were days when I receive incomplete packages.’ (Participants 11)

**Subcategory 2.1.3: Sharing of the food supplies:** Even though the ready-to-use therapeutic food and the food by prescription were prescribed as therapy for the sick child only, some participants reported that it was used as a meal for the family. In some families, the ready-to-use therapeutic food was used as butter, while some were allowing sharing with the rest of the children in their families (Participants 3 and 9). Only two of the study participants reported that they used to sell their food packages when they felt desperate for money (Participants 3 and 12). Their words were depicted as follows:

‘The children used to share the food therapy with the rest of the children within the household and those that have come to play with them.’ (Participant 3)

‘The food packages were very helpful, but at times I used to be desperate for money, such that I used to sell them along the way home.’ (Participant 12)

#### Category 2.2: Health education

The health education was imparted to each patient before they were consulted to promote good public health practice and shaping the behaviour of patients. It promotes all basic information about the condition or care rendered within a very short time (Sharma 2016). Table 1 illustrates one subcategory that was identified, which was health education too long.

**Subcategory 2.2.1: Health education too lengthy:** Even though all the study participants were happy with the health education they received, they also complained that the health education was too long and difficult to remember. The statements of the participants were recorded as follows:

‘The health education used to be long and cumbersome … Just imagine you are a lay-person, and they expect you to learn about HIV, TB, and Malnutrition in one session. The session becomes too long, such that I lose concentration.’ (Participant 1)

‘[*W*]ith all these long health education and medical language. We cannot cope.’ (Participants 3, 8 and 10)

## Discussion

The study explored and described the experiences and practices of the caregivers regarding the children’s nutritional needs during TB treatment at BCMCF. The key findings that emerged included the nutritional situation at home and the health facility’s nutritional support. Both themes elicited the importance of a balanced diet to achieve proper nutrition for children diagnosed with TB. The experiences and practices of the participants are congruent with literature, which indicates that lacking a balanced diet can result in malnutrition, which is responsible for twice the number of TB cases compared with HIV globally (Nair et al. 2017). Literature further reveals that there are maternal sociodemographic and medical factors that can affect exclusive breastfeeding (UNICEF 2018). According to the study that was conducted in Addis Ababa by Sahile, Tezera, Haile Mariam, Collins and Ali (2021) on nutritional status and TB treatment outcomes, undernutrition was found to be associated with an increased risk of mortality and relapse in people with TB disease. The authors recommended strengthening nutritional assessment, counselling and interventions as a standard practice for TB patients. Some of the sociodemographic factors include the age of the mother, employment status and knowledge about the benefits of exclusive breastfeeding (Kandeel et al. 2018).

The nutritional situation at the place of residence for a child treated for TB is one of the major concerns within a healthcare system (American Academy of Pediatrics 2019). An optimal level of good nutrition is essentially a successful TB treatment across all TB patients (WHO 2018). Thus, the combination of both malnutrition and childhood stage is mostly likely to be accompanied by worst TB outcomes (Munsi et al. 2017). Most of the children get TB infection from their family members because of their vulnerable immune system, which can be exacerbated by poor nutrition. The new TB cases among children are a sign of a sentinel event and continuous infection in their place of residence or community (Ki & Shingadia 2017). Another study conducted in Eswatini indicated that most of the children diagnosed with TB had their parents on TB treatment or having TB symptoms (Baylor College of Medicine Children’s Foundation – Swaziland 2015–2016). Most of the caregivers of the children diagnosed with TB get diagnosed with TB post a reverse contact tracing, whereby after diagnosing a child with TB all the family members are screened for TB and/or investigated about the death of family members from an unknown cause (Becker et al. 2020; Mandalakas et al. 2017).

An evaluation of the quality of care rendered towards children diagnosed with TB in Uganda, a similar setting like Eswatini, indicated prescription and provision of ready-to-use therapeutic food to malnourished children as essential to achieving favourable TB outcomes (Wanzira et al. 2018). A study conducted in 2019 at Eswatini to assess the barriers to adherence to antiretroviral therapy found that hunger, unemployment and scarcity of financial resources are of high concern among families. Agricultural activities can also be promoted among all families in the country to eliminate hunger among families (National Disaster Management Agency 2016).

The National Disaster Management Agency (NDMA) of Eswatini acknowledges that some TB clients including children are deprived of full access to food by prescription or ready-to-use therapeutics supplies because of the high demand that the institution could not meet (Musonda 2020).

Raru et al. (2022) assert that the magnitude of stunting and wasting are relatively high in Ethiopia, which indicates that undernutrition is a public health concern among children. In addition, region, place of residence and age of the child were significantly associated with stunting, and region, household wealth index and age of the child were significantly associated with wasting. Therefore, improving the socioeconomic status of households through economic empowerment would help to decrease the prevalence of malnutrition among children under the age of 5 years. Atkins et al. (2022) attest that TB has been repeatedly shown to have socioeconomic impacts in both individual level and ecological studies; however, much less is known about this among children and adolescents and the extent to which being affected by TB during childhood and adolescence can have life-course implications. Therefore, despite the negative impact on children’s caregivers regarding paediatric nutrition during TB treatment, possible solutions to compensate for or reverse this are of importance.

### Limitations of the study

The study was limited to caregivers of children aged 0–15 years who were treated for TB disease at BCMCF-SD at Mbabane. Therefore, the researchers conducted purposive sampling and validated the data using a paper-based TB register. Only the semi-structured interviews were conducted. The researchers decided to interview caregivers of children treated in the certain periods because caregivers of children treated more than 2 years ago would have forgotten critical information that was needed during the interviews. Also, this study was only conducted in one Baylor College of Nursing Children’s Foundation – Eswatini.

### Recommendations

The findings of this research article necessitate a number of strategies that need to be put in place to address malnutrition among children diagnosed with TB. Some of the strategies include the promotion of entrepreneurship and vocational training to alleviate poor nutritional situation at household level. Agricultural activities can also be promoted among all families in the country to eliminate hunger among families. Reduction of hunger will also reduce the number of families that share the food parcels of ready-to-use therapeutic food meant for the sick child with the rest of the children in the family or from the neighbourhood. Therefore, the nutritional support for children diagnosed with TB needs to be prioritised. In relation to the nutritional support at health facility level, the research article recommends that the health facility needs to revisit the time taken for health education and ensure that all stocks for food by prescription are well ordered in time.

The findings also provided evidence-based information to help both the caregivers and the HCWs in the quality management of children diagnosed with drug-susceptible TB and malnutrition. Policies aiming to address malnutrition and drug-susceptible TB in children should consider the findings of the study to ensure an improved and standardised quality of care offered to malnourished children enrolled in TB treatment. Authorities should provide a platform for all relevant healthcare providers and the caregivers to undergo regular trainings and attend workshops to update themselves for combating both malnutrition and tuberculosis.

## Conclusion

The main findings of the study are home nutritional situation of the child, the social environment, health facility’s nutritional support and too lengthy health education. Remaining underweight during TB treatment is associated with a higher risk of unsuccessful TB treatment outcomes and relapse. Caregivers require comprehensive programmes that will empower them to render effective care to children diagnosed with drug-susceptible TB and malnutrition. Programmes should focus on working together with caregivers and HCWs to develop a conducive PSS environment for both the caregiver and the affected child to improve the success rate of TB treatment. Therefore, screening for TB in children is important, as it is one of the leading causes of death worldwide. Most children who die from TB are never diagnosed or treated. Screening may be useful to identify children with possible TB and refer them for further testing. Malnutrition, also as a global concern and particularly in children, negatively impacts mortality, morbidity, educability and productivity of millions of children. Therefore, it is important to combat malnutrition by eradicating extreme poverty and hunger. Health education and health promotion should be conducted at all health facilities with regular intervals, as well as within the communities. These initiatives could improve healthcare practices in both home environment and healthcare facilities of Eswatini and other countries of the world.
